# A Conversational Agent (PracticePal) to Support the Delivery of a Brief Behavioral Activation Treatment for Depression in Rural India: Development and Pilot-Testing Study

**DOI:** 10.2196/73563

**Published:** 2025-08-29

**Authors:** Ravindra Agrawal, Kimberley Monteiro, Nityasri Sankha Narasimhamurti, Shreya Sharma, Amruta Suryawanshi, Aman Bariya, Shravani Narvekar, Lilianna Bagnoli, Mohit Saxena, Lauren Magoun, Shradha S Parsekar, Julia R Pozuelo, Neal Lesh, Mohit Sood, Tanushri Sharma, Harshita Yadav, Anant Bhan, Abhijit Nadkarni, Vikram Patel

**Affiliations:** 1 Sangath Panaji India; 2 Department of Psychiatry Manipal Hospital Panaji India; 3 Antarman Centre Panaji India; 4 Impact Lab Dimagi India New Delhi India; 5 Dimagi India New Delhi India; 6 Dimagi United States Cambridge, MA United States; 7 Department of Global Health and Social Medicine Harvard Medical School Boston, MD United States; 8 Sangath Bhopal India; 9 Department of Population Health London School of Hygiene & Tropical Medicine London United Kingdom; 10 Addictions & Related Research Group Sangath Panaji India; 11 Department of Global Health and Population Harvard TH Chan School of Public Health Boston, MD United States

**Keywords:** conversational agent, chatbot, digital tool, homework assignment, behavior activation, treatment adherence, depression, psychological treatment

## Abstract

**Background:**

Brief psychosocial interventions, such as the Healthy Activity Program (HAP), which are based on behavioral activation and delivered by nonspecialist providers (NSPs), have emerged as cost-effective solutions for the treatment of depression. HAP treatment outcomes are improved by the engagement of patients in activation-focused homework assignments and their adherence to these assignments during therapy. Currently, patients are expected to complete these homework assignments using a paper workbook.

**Objective:**

The aim of this study was to describe the user-centered development process of PracticePal, a chatbot designed to enhance patient engagement and homework adherence, and to evaluate its feasibility and acceptability as a therapy aid in India.

**Methods:**

We used a user-centered approach to co-develop PracticePal, incorporating conversational flows and video scripts in Hindi. The chatbot was piloted with 30 participants having depression who were receiving the HAP from 15 nonspecialist counselors in primary care in rural Madhya Pradesh, India. The feasibility and acceptability of PracticePal were assessed through engagement data, in-depth interviews with a subset of 6 participants, and focus group discussions with 11 counselors. Treatment completion rates and changes in depressive symptoms were explored as secondary outcomes.

**Results:**

Average patient engagement spanned 29 days (95% CI 24-34) during the 60-day treatment period. The engagement of patients with PracticePal increased as their treatment progressed, particularly after the third HAP session. Of the 30 patients, 20 (67%) accessed more than half of the multimedia content available on the chatbot. On average, there was a greater frequency of self-initiated engagement (1558 out of a total of 1835 times, 84.9%) across all sessions compared with reminder prompts (277 out of 1835 times, 15.1%). All 30 patients completed treatment and experienced a reduction in the mean Patient Health Questionnaire-9 score from 13 (95% CI 12.6-13.6; signifying moderate severity) to 4 (95% CI 2.9-4.7; signifying none/minimal severity). Patients found the chatbot’s reminders for activities, mood tracking, and video messages helpful and observed that it could help others in their social network. NSPs also reported improved participation of patients in the homework tasks compared with the paper workbook. A few patients faced challenges with low internet bandwidth, and those with limited literacy suggested increasing the amount of video content for easier accessibility.

**Conclusions:**

The PracticePal chatbot is a feasible and acceptable therapy aid to complement a psychological treatment, with promising potential to enhance the effectiveness of NSP-delivered psychosocial interventions in low-resource settings. Future steps include conducting a fully powered randomized controlled trial to assess its effectiveness in improving mental health outcomes.

## Introduction

Depression is a common mental health issue, with symptoms, such as low mood, fatigue, loss of interest, and insomnia, as well as unexplained somatic symptoms. According to the World Health Organization, 5% of adults currently experience depression [1]. As of 2023, an estimated 280 million individuals worldwide had depression, including 56 million living in India (approximately 4.5% of the country’s population) [2]. These numbers are compounded by a large treatment gap [3], especially in India [4], where 85% of people with depression do not receive an evidence-based intervention for their illness. This is largely due to the severe shortage of mental health professionals, as there are only 0.03 psychologists and 0.20 psychiatrists per 10,000 individuals [5]. Task sharing, which involves training nonspecialist providers (NSPs) to provide low-intensity mental health treatments, has been found to be effective and cost-effective for the treatment of depression [6-9].

The Healthy Activity Program (HAP) is one such evidence-based psychological intervention delivered by NSPs in routine primary care settings [10], and it is already being scaled up in partnership with health care systems [11]. The core active ingredient of HAP is behavioral activation, which is based on the principle that engaging in specific activities can help improve mood and counteract the patterns of avoidance, withdrawal, and inactivity that often accompany depression [12,13]. The therapy focuses on identifying and scheduling meaningful and enjoyable activities that align with an individual’s values and interests, with the goal of increasing positive reinforcement from the environment. The HAP, which is delivered in 6 to 8 sessions, includes the assignment of homework to keep clients engaged between sessions and offers them opportunities to monitor their mood and to practice and ultimately master the skills learnt during treatment. Typically, homework assignments are completed using paper-based worksheets or pamphlets. However, in rural and low-resource settings, distributing and completing printed materials pose logistical and literacy challenges. In addition, these printed materials lack interactivity and tailored reminders, and require privacy, which may be difficult for individuals living in crowded family environments. Therefore, we aimed to explore whether the deployment of digital technologies might help address these challenges, after being inspired by research showing that such tools are effective in improving health outcomes in low-resource settings [14-16].

Conversational agents (CAs) are digital tools that interact with users through text or voice (eg, Siri and Alexa). CAs have the potential to increase the availability of mental health support and reduce its costs [17]. One type of CA is a rule-based chatbot, a software designed to simulate a human conversation through a scripted conversation workflow and text-based communication. Chatbots provide a promising solution for increasing patient engagement, offering personalized and interactive support, and filling gaps in traditional mental health care systems. With their ability to deliver tailored content and reminders, chatbots can enhance the effectiveness of interventions [7,14].

However, most existing CAs operate in English and have been primarily tested in high-income and resource-rich countries [10,17]. To address this gap, this study deployed user-centered and participatory action research methods to design “PracticePal,” a rule-based chatbot intended to serve as a therapy aid in the delivery of the HAP by NSPs, such as accredited social health activists (ASHAs), who are on-ground community workers of India’s National Health Mission. The chatbot was designed to support engagement between therapy sessions by prompting patients to complete their homework assignments and mood ratings, and reinforce therapeutic messages via engaging multimedia content. In this paper, we describe the development process of PracticePal and a pilot study evaluating its feasibility and acceptability among patients receiving a brief psychological treatment for depression in a rural, underserved setting of central India.

## Methods

### Ethical Considerations

This study was approved by the Institutional Review Board at Sangath (approval letter number: RA_2023_89) and the Indian Council of Medical Research. The approval provided by the institute’s Institutional Review Board and by the Indian Council of Medical Research is for study implementation, data collection, and data analysis. Informed consent was obtained from all study participants (ie, ASHAs and participants with depression) to be part of the study and to allow the collection of quantitative and qualitative data for further analysis. All participants were assured that their data would be deidentified and that their identities would be kept confidential. The patient participants were provided smartphones by the study team for the duration of the study for two main reasons: (1) to ensure that their engagement was not influenced by restricted access (as many participants had only 1 smartphone per household) and (2) to collect research data in anonymized form. Many of the participants who owned a smartphone also had the Telegram messaging app (Telegram LLC) already downloaded and registered in their own name. This would have meant that their identity would be visible at the time of collecting research data from the TextIt backend (a cloud-based platform that allows users to create and manage interactive messaging workflows). The study-provided phone ensured registration on the Telegram app with an anonymized participant ID. No monetary compensation was provided to the participants for taking part in the study, although they were provided with light refreshments, such as tea and snacks, following the interviews. The manuscript does not include any identifiable images of individual participants.

As this was an early-stage pilot study focused on assessing the feasibility and acceptability of a digital intervention in a real-world context, which had a small sample size and was nonrandomized, it was not prospectively registered.

### Setting

The study was conducted in the Raisen district of Madhya Pradesh, India. Madhya Pradesh is one of the largest states in India, and is classified as one of its least developed states, with a predominantly rural population [18-21]. The lack of access to mental health services in the state has made it a key priority for the health system [22,23]. However, smartphone penetration is expanding at a rapid pace, with the state having added nearly 2.2 million new subscribers between May 2023 and February 2024 [24,25].

### Sample

In the pilot study, the HAP was delivered by ASHAs as part of a community mental health program led by Sangath, in collaboration with the local government. ASHAs are female community health workers who serve as incentivized volunteers in the government health system. Typically, each ASHA serves a population of around 1000, primarily in rural areas, with key responsibilities focused on maternal and child health, and serves as a vital link between the community and the health system [26-28]. The ASHAs were trained in delivering the HAP by Sangath’s trainers. Recruitment was conducted by the ASHAs at the residences of the study participants. The study participants were conveniently sampled during routine home visits by the ASHAs from eligible adult residents of the study sites. Eligible study participants were above 18 years of age, were fluent in Hindi, screened positive for depression on the Patient Health Questionnaire-9 (PHQ-9; score of ≥10), and provided written informed consent to be a study participant. We aimed to achieve maximum variability in gender, age, and education level in our sample. Additionally, a subsample of ASHAs who provided the counseling participated in 2 focus group discussions (FGDs) to share their perspectives on using the chatbot as a therapy aid.

A wider range of stakeholders was recruited during the development phase, including other ASHAs, a range of HAP practitioners, and members of the community. The objective of involving multiple stakeholders was to gather diverse perspectives, triangulate the information collected from these diverse stakeholders, and ensure that the chatbot was culturally appropriate and acceptable within the community.

### Study Design

**Figure 1:**
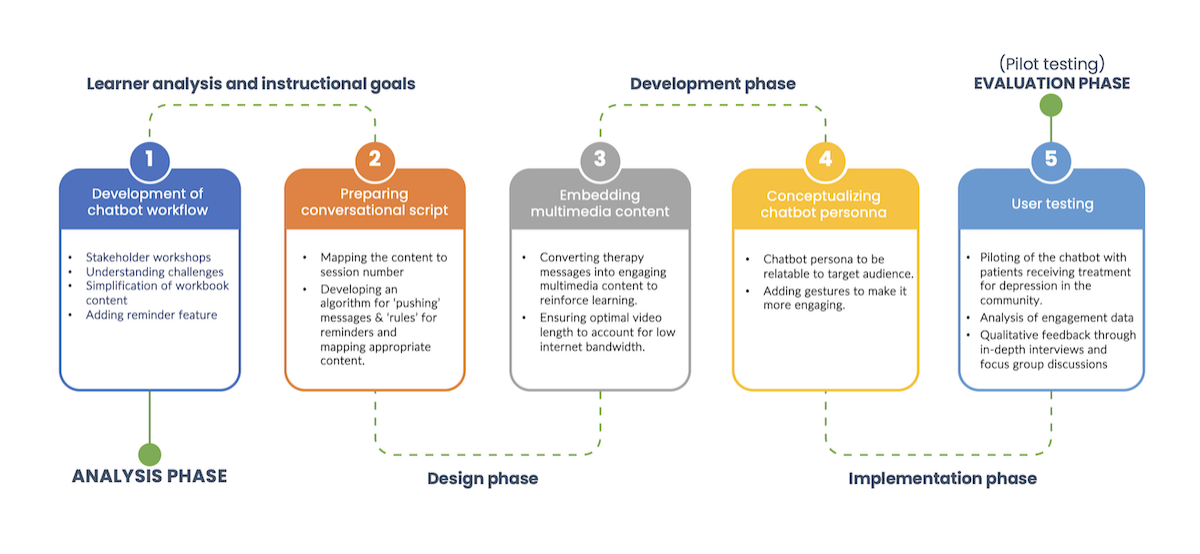
Steps in the development of the PracticePal chatbot.

We developed PracticePal in partnership with Dimagi, a software social enterprise specializing in scalable digital solutions for frontline workers in low-resource settings. We used an iterative approach to design and develop the PracticePal chatbot, guided by the ADDIE (Analysis, Design, Development, Implementation, and Evaluation) framework [29] and by previous digital intervention development projects in mental health [30-32]. User-centered development was carried out in 5 phases: analysis, design, development, implementation, and evaluation. The phases of development are outlined in Figure 1 and described in the sections below, with each phase building upon the findings of the previous one.

#### Analysis

The objective of this phase was to identify the challenges with the paper workbook, assess the feasibility of the chatbot, and define its key features. We conducted 3 stakeholder workshops, involving HAP practitioners (n=5), ASHAs (n=17), and chatbot developers (n=3). During the workshops, we identified several key challenges associated with using the paper workbook, including the need for careful storage, its lack of privacy, and its tendency to be lost or damaged. For patients with depression, completing the workbook felt burdensome due to low energy and motivation. To simplify the content, the workbook had to be less detailed, limiting the depth of therapy messages. Importantly, there were no reminders to do the homework tasks. Providers also expressed a need for real-time updates on their patients’ homework progress and mood ratings, and a way to reinforce therapy messages between sessions. These findings shaped the design of the chatbot, focusing on simplifying patient engagement, enhancing privacy, and integrating reminders and multimedia content to support therapy.

#### Design

The objective of this phase was to use the findings from the analysis phase to design the chatbot features and components, with a focus on developing a conversational flow that aligned with the therapeutic progression of HAP sessions. Following the first therapy session, users gained access to psychoeducational content related to that session’s topics. After the second session, users could access all chatbot features, including activity scheduling, rescheduling, and additional multimedia content. The multimedia content was designed to reinforce the core messages of the HAP, and it was designed in consultation with stakeholders. The videos were scripted and recorded in Hindi, were deliberately kept brief (under 5 minutes) based on suggestions from experienced HAP practitioners, and were kept accessible multiple times (a detailed description of each video can be found in Multimedia Appendix 1). Since the HAP is designed to be delivered by NSPs (ie, ASHAs in this study context), it was essential for the chatbot to maintain fidelity to the HAP counseling manual. This required the chatbot’s conversational messages, the multimedia content, and the features, such as scheduling activities, mood ratings, and homework tracking, to be carefully structured to mirror the patients’ stages in therapy. All patient participants received the full course of psychological treatment, consisting of 6 sessions. At any given point during the study period, participants could be at different stages of therapy, depending on their individual scheduling preferences. However, all participants ultimately completed all 6 sessions. Participants were encouraged to enter their session number while engaging with the chatbot during the intersession period, which allowed the chatbot to provide access to content and features relevant to that session (managed via a backend algorithm, as explained in Multimedia Appendix 2) and helped the study team to map the frequency and duration of chatbot engagement to the corresponding therapy sessions when collecting data from the backend. Another key aspect was to design an avatar that would be relatable to users, be more engaging, and improve trustworthiness through humanization [33]. Accordingly, the chatbot’s avatar was called *Mann-mitra* (which, in Hindi, means a friend of the mind or mood). NSPs and members of the community gave suggestions on its characteristics, recommending that the avatar look like a married woman dressed in a saree, like ASHAs. To increase user engagement, the avatar was also given various gestures and expressions depending on the status of task completion (see Multimedia Appendix 3).

During this phase, we also determined other key components of the chatbot, such as its overall esthetics, its technical specifications, and the frequency of reminders sent to participants. All participants received activity-specific reminders at the beginning of the day (ie, 10 AM) on which the activity was scheduled. Based on feedback received during stakeholder meetings, additional reminders were sent at 5 PM, 7 PM, and 9 PM.

#### Development

Over a period of 8 months, from October 2022 to May 2023, we added features allowing clients to schedule their activities, set reminders, log activity (homework) completion, record their mood, and access multimedia content. The therapy messages were packaged into engaging multimedia content, while, at the same time, we were mindful to keep the length of the videos between 2 and 5 minutes to ensure optimum attention span and reduce file size. We adopted the principles of the agile project management approach [34] by developing a session-wise workflow and adding content incrementally, with features added based on the feedback received from stakeholders. Multiple versions of the chatbot were tested to identify errors in the algorithm, and the nodes were pruned to enable triggering of an appropriate “decision point.”

Screengrabs of the chatbot interface are provided in Multimedia Appendix 4. PracticePal was developed on the TextIt platform and launched on Telegram, a messaging platform. We used Telegram as a channel for this chatbot due to the lower cost associated with it (compared with other messaging platforms). After development, the chatbot underwent 2 months of rigorous testing by the project team to identify and resolve bugs.

**Figure 2:**
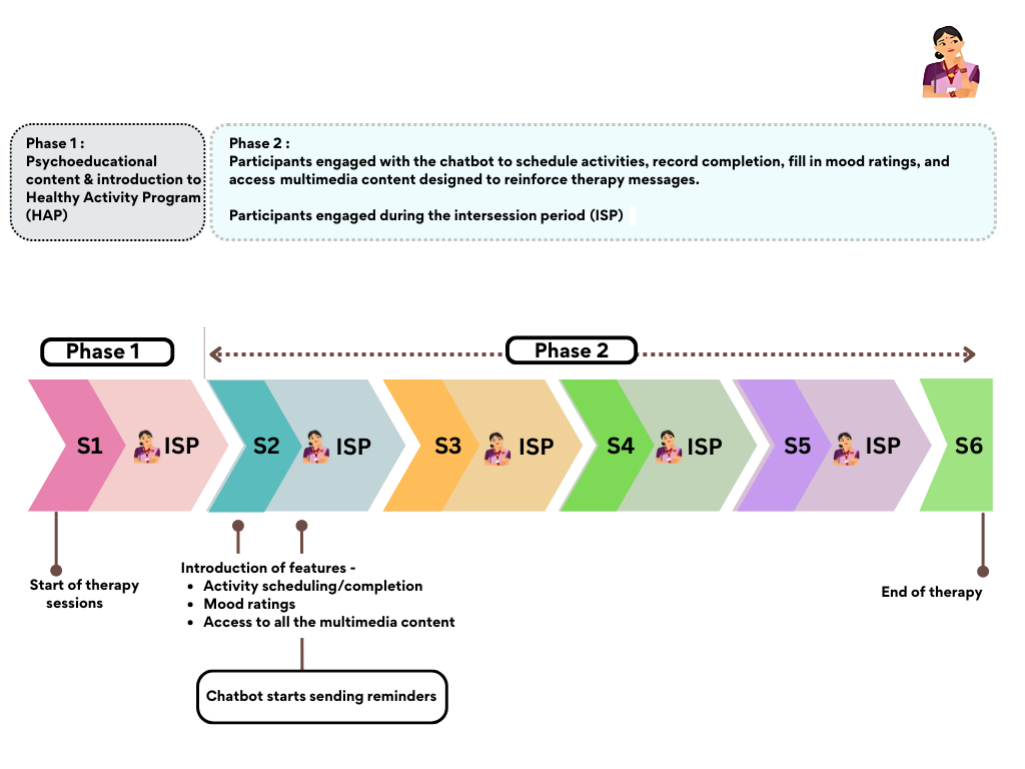
Phase-wise outline describing the PracticePal chatbot features for patients. S: counseling session.

The PracticePal chatbot was piloted in a single-arm, nonrandomized study in August 2023 in the Raisen district of Madhya Pradesh. Trained ASHAs, who were already delivering the HAP to patients with depression, introduced the chatbot during the first counseling session, guiding patients on how to use its features. All subsequent counseling sessions were conducted at the residences of the study participants. ASHAs received weekly summaries on each patient’s engagement, homework completion, and mood ratings, which helped them prepare for subsequent sessions. They also addressed any technical or content-related issues during the therapy, ensuring patients fully benefited from the digital tool. This summary was shared either as a printout or as a PDF sent to their smartphones. The process of integrating the chatbot during the therapy has been summarized in Figure 2.

#### Evaluation

To evaluate the usability, feasibility, and acceptability of PracticePal, we collected both quantitative and qualitative data. Quantitative data on engagement were extracted from the backend of the chatbot platform, which tracked the frequency of access, total time spent engaging with the chatbot (including watching the multimedia content), and the depth of engagement (measured in terms of completion of homework within the workflow). The patient baseline PHQ-9 and endline PHQ-9 assessments were conducted by an independent researcher. Endline assessments were carried out through a phone survey. We also carried out purposive sampling to select patients and ASHAs, with whom we conducted postintervention in-depth interviews (IDIs) and FGDs, respectively, to gather qualitative data on user experience, perceptions of the chatbot, and suggestions for improvement. The interview guide was designed to achieve the study objectives related to feasibility (the extent to which this digital innovation could be successfully used or implemented within a given setting) and acceptability (perception among stakeholders that the use of the chatbot is agreeable, palatable, or satisfactory) [35]. Patients were asked about their experiences using the chatbot, including ease of engagement, scheduling homework tasks, completing mood ratings, helpfulness of reminders, and understanding the therapy messages. ASHAs were asked about their experiences delivering therapy to patients who used the chatbot and their perceptions of its impact on homework completion rates. The IDIs and FGDs were conducted by Kavita Mandhare, an independent female researcher with a master’s degree in clinical psychology and more than 4 years of experience in qualitative research methods. She was assisted by the project staff in identifying the participants and ensuring their availability for the IDIs or FGDs. First contact with the participants was over a phone call, following which the IDIs and FGDs were conducted in person in the local vernacular language (Hindi). Six IDIs were conducted with patients aged 21-43 years (5 female patients and 1 male patient; 5 married and 1 unmarried) either at each patient’s house or at the house of a trusted friend. Four of these patients were homemakers, and 1 was a student. The education level ranged from 10th grade to graduate. Two FGDs (FGD1: n=6; FGD2: n=5) were conducted at the nearest health centers. In both scenarios, every effort was made to ensure the privacy of the participants, and they were informed that the researcher was independent from the intervention delivery team and was collecting feedback for improvement purposes. No participant refused to participate or backed out. All interviews were audio-recorded, lasted between 45 and 60 minutes, and were translated and transcribed into English. Field notes were taken during and after the interviews, and they guided the analyses.

### Analysis

The quantitative data of all study participants were extracted by 2 study team members (AS and KM) and analyzed using descriptive analysis. The evaluation focused on several key metrics, including (1) the sociodemographic profile of the participants; (2) engagement with the chatbot, through the following key metrics: (a) frequency of interactions, (b) duration of interactions, (c) task completion (eg, scheduling activities, homework completion, and submitting mood ratings), (d) viewing of multimedia content, (e) user retention, and (f) qualitative feedback; and (3) patient outcomes as measured through treatment completion rates and changes in depressive symptoms (measured by the PHQ-9 score). We conducted an independent *t* test to assess for significant changes in PHQ-9 scores and multiple regression analysis to examine the relationship of gender and age with chatbot engagement. Qualitative data were analyzed using the thematic analysis approach with the NVivo software (version 14; Lumivero). A deductive approach was employed to explore key aspects of acceptability and feasibility based on participants’ experiences and reports. The researchers first created initial codes from the transcripts, which were then grouped into themes and subthemes according to the original research questions and the interview guide. The reliability of the coding system was tested by carrying out blinded, double coding of the first 2 transcripts by SS and KM to reach a consensus on the preliminary codes and themes. While the transcripts were not returned to participants for comment or correction, the analysis team held regular peer discussions and reviewed the codes and themes in an ongoing way. Formal participant validation of the findings was not done, but care was taken to include different and contrasting views, such as minority or dissenting opinions, in the final analysis. The meta categories for coding were (1) acceptability of the chatbot, (2) feasibility of the chatbot as a therapy aid, and (3) challenges. The researchers developed a comprehensive codebook that was reviewed and finalized by RA and NSN. SS and KM subsequently coded the remaining transcripts and generated higher-order themes and subthemes. Finally, a narrative interpretation of the themes was developed, and illustrative quotes were selected. Neither of the coders was directly involved with the intervention delivery at the study site.

## Results

### Participant Characteristics

Our study sample included 30 patient participants. The majority of the participants (23/30, 77%) were married, and 57% (17/30) were women. Most participants (23/30, 77%) were between 18 and 35 years of age (median 27.4 years). The educational backgrounds of these 30 participants showed considerable variation. Of the 30 participants, 7 (23%) had completed primary education (up to class 4), 15 (50%) had studied up to high school (up to class 10), 3 (10%) had completed higher secondary education (up to class 12), 3 (10%) were graduates, and 2 (7%) held postgraduate degrees. All participants were familiar with the use of a smartphone and were able to operate the study-provided mobile phone.

### Participant Engagement

All 30 participants interacted with the chatbot between sessions 1 and 6 over the 60-day treatment period, exhibiting varying levels of engagement.

#### Frequency and Duration of Use

**Figure 3:**
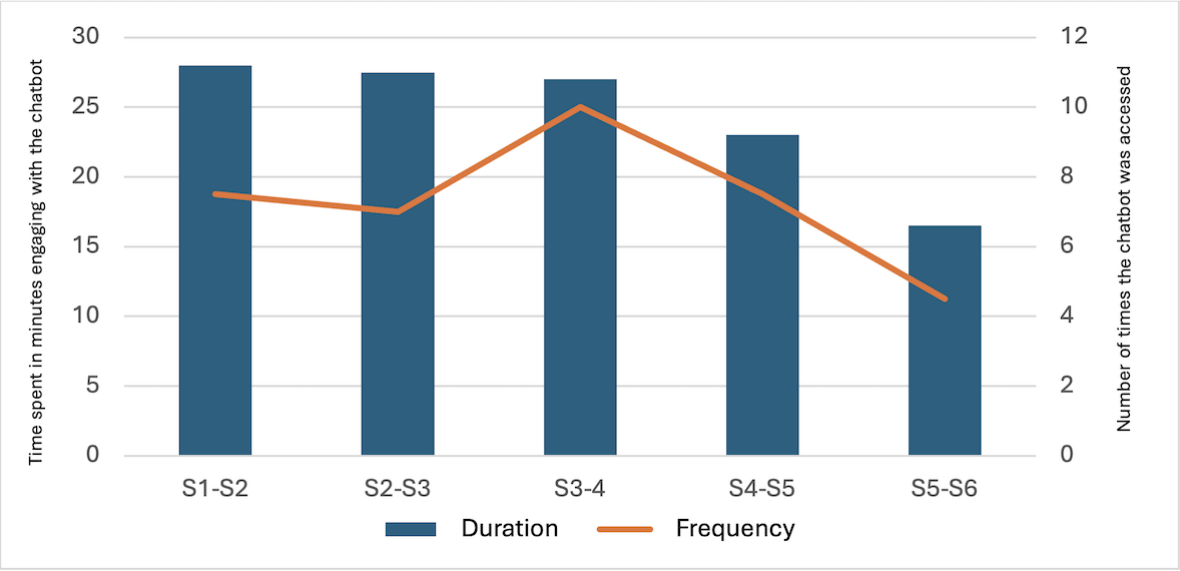
Overall frequency and duration of engagement during therapy with the chatbot.

We found that a participant engaged on average 53 times (95% CI 38.44-67.69) with the chatbot and spent an average of 233.5 minutes (ie, 3 hours and 53.5 minutes; 95% CI 119.74-347.33) engaging with the chatbot during the entire 60-day treatment period. In between any 2 therapy sessions, the average frequency of engagement was 10.61 times (95% CI 7.69-13.54), and the time spent was 46.7 minutes (95% CI 23.95-69.47). Figure 3 graphically represents the engagement frequency and duration. Analysis of chatbot engagement revealed a robust interaction immediately following session 1, when the chatbot was introduced. To account for varying participant engagement, median values were used. Between sessions 1 and 2, participants engaged with the chatbot on average 7.5 times for 28 minutes. As expected, engagement increased after session 2, with the introduction of features like activity scheduling and mood ratings. However, engagement gradually decreased to a median of 4.5 times and 16.5 minutes between sessions 5 and 6. Notably, all 30 (100%) participants interacted with the chatbot during sessions 1-5, with a slight decrease to 93% (28/30) after session 5. During the intersession periods, a high proportion of participants (29/30, 97%) engaged with the chatbot for over 10 minutes, with more than half (18/30, 60%) interacting for 20 minutes or longer.

#### Participant Use of the Chatbot’s Features

**Figure 4:**
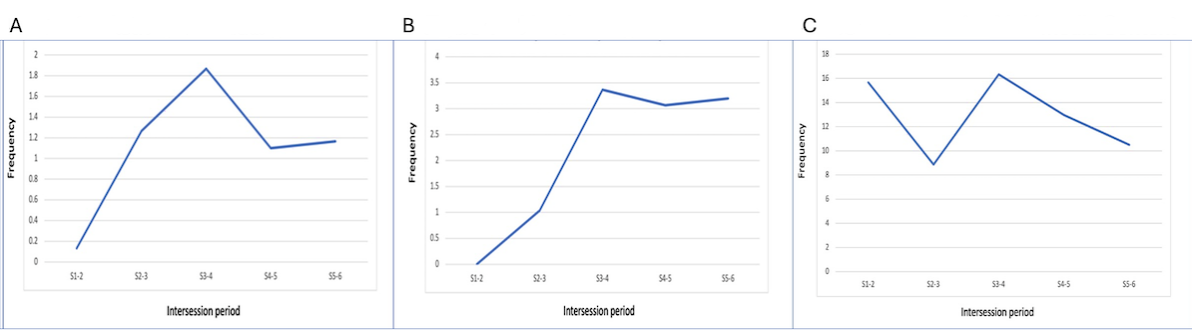
Participant engagement with PracticePal's features: (A) activity scheduling feature; (B) mood ratings; and (C) accessing multimedia. S: counseling session.

A key function of the chatbot was to encourage participants to schedule and engage in behavior activation tasks (homework activity) during the intersession period. Participants received reminders to schedule and complete the homework, record mood ratings, and access multimedia content. These reminders were sent at 10 AM, 5 PM, 7 PM, and 9 PM on the day an activity had been scheduled, irrespective of whether the individual had been active on the chatbot that day or not. All participants scheduled at least one homework activity and recorded mood ratings across sessions. Nearly two-thirds (22/30, 73%) of participants accessed more than half of the multimedia content presented by the chatbot. Figures 4A, 4B, and 4C depict the average engagement of the user with the chatbot’s features of scheduling homework activities, reporting mood, and accessing multimedia content during the intersession period, respectively.

While the multimedia content was accessed at a high rate throughout the therapy duration, the use of features, such as activity scheduling and reporting of mood, picked up as the therapy progressed. This trend may reflect delayed understanding and orientation to the chatbot’s features and was also reported during the IDIs.

#### Self-Initiated Versus Prompted Engagement

**Figure 5:**
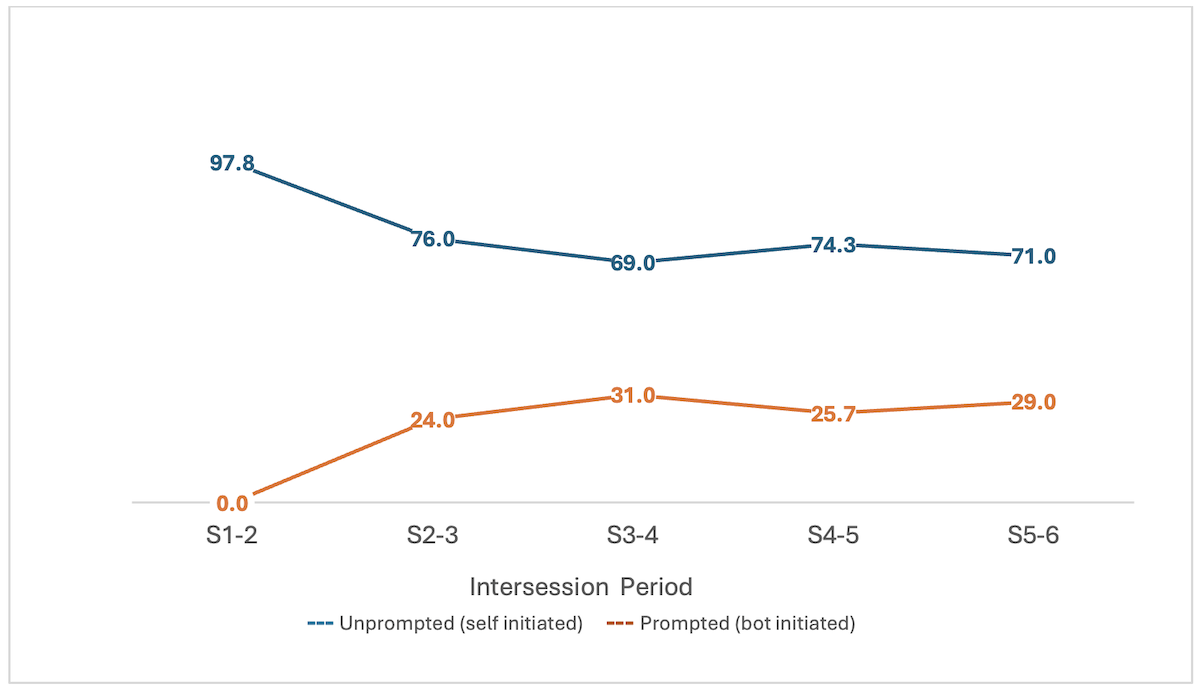
Percentage of overall self-initiated (unprompted by chatbot) and chatbot-initiated (prompted by chatbot) engagements with the PracticePal chatbot between each therapy session. S: counseling session.

Participants engaged with the chatbot either through self-initiated interaction (unprompted) or in response to chatbot reminders (prompted). The chatbot reminders started only after session 2 and were restricted to 4 per day. We defined self-initiated engagement as the use of the chatbot by the patient either without any reminder or more than 1 hour after a reminder had been sent. Engagement within 1 hour of receiving a reminder was considered prompted. Self-initiated access (1558 out of a total of 1835 times, 84.9%) was substantially higher than prompted access (277 out of 1835 times, 15.1%) across all participants, indicating that most participants engaged with the chatbot voluntarily, beyond reminders (Figure 5).

#### Pattern of Engagement Based on Sociodemographics

On average, women accessed the chatbot with greater frequency and tended to spend twice the amount of time engaging with it compared to men. Similarly, younger individuals between the ages of 18 and 25 years had frequencies and durations of engagement that were 2-3 times those for patients aged above 35 years. Table 1 lists the various engagement metrics in terms of gender and age.

**Table 1:** Sociodemographic differences in the pattern of engagement with the chatbot.

Sociodemographics		Frequency of engagement (times), value (95% CI)	Time spent engaging (minutes), value (95% CI)
**Gender**			
	Female	61.88 (36.57-87.20)	62.24 (22.76-101.71)
	Male	41.54 (32.57-50.51)	26.4 (15.40-37.40)
**Age (years)^a^**			
	18-25	71.78 (27.72-115.84)	66.58 (10.54-122.62)
	26-35	50.57 (33.35-67.79)	42.78 (26.61-58.94)
	>35	34.00 (22.95-45.05)	22.2 (19.53-25.26)

^a^Regarding age, the *P* values were .15 for frequency of engagement and .32 for time spent engaging.

#### Retention Rate

Despite decreases in the frequency and duration of interactions over time, participant engagement with the chatbot remained remarkably high throughout the treatment period. All 30 (100%) participants actively engaged with the chatbot from session 1 to session 5, and there was a slight decline in engagement observed after session 5, with 28 (93%) participants still engaging with the chatbot at the end of treatment.

### Participant Outcomes

All the participants in the pilot study completed the treatment. The average PHQ-9 score at baseline was 13.2 (95% CI 12.53-13.87) and decreased significantly to 3.8 (95% CI 2.77-4.83) upon treatment completion (*P*<.001). This reflects a reduction from a clinically moderate range of severity to a clinically nonsignificant range. Multiple regression analysis examined whether gender and age had an effect on the frequency of engagement. While the results showed that females engaged with the chatbot at a higher frequency, it was statistically insignificant (*P*=.19). A negative correlation was seen between age and the frequency of engagement, that is, as participant age increased, their engagement frequency decreased by 1.85 units, a relationship that was marginally significant (*P*=.07) (see Table 2).

**Table 2:** Multiple regression analysis to examine the relationship of the gender and age of patients with chatbot engagement.

Independent variable^a^	Regression coefficient	Standard error	*t* test (*df*)	*P* value
Gender (male)	–18.26	13.61	–1.34 (27)	.19
Age (years)	–1.85	0.98	–1.89 (27)	.07

^a^*R*^2^=0.1768 (model explains 17.7% of variance in engagement); adjusted *R*^2^=0.1159; residual standard error=36.83; *F*-statistic=2.9 on 2 and 27 degrees of freedom; model *P* value=.07 (not statistically significant at the 5% level).

### Acceptability, Feasibility, and Effectiveness of PracticePal: Qualitative Findings

#### What Participants Liked About PracticePal

Participants generally found the PracticePal chatbot to be a valuable tool. They appreciated how the chatbot simplified concepts and made it easier for them to understand the principles of behavior activation, as explained by the ASHAs during the HAP therapy sessions. It reinforced the therapy messages and helped in the application of behavior activation strategies.

She (ASHA counsellor) told me, I will get a lot of help through this mobile if I go through it. She asked me to go through it and I did. Madam (ASHA counsellor) told me about social interaction, and I saw that (on the chatbot); it helped me a lot.

42 years, male, married

Because if you keep it (mobile) then you also get entertainment also. It (chatbot) guides us on everything, such as, if we have tension then take our mind off it, meet people, talk to them. It tells about whatever problems we have. I used to listen to and watch the videos every day.

30 years, female, married

The multimedia content was a well-received feature. Participants found the videos relatable and reported that they summarized and reiterated the session learnings effectively.

In the video they used to explain it so nicely that if we are in depression then we should walk outside so that our mind will be light, all this they explained properly. So, in that I understood a little that I should not be at home, I should go outside, roam, see others a little, understand everything.

22 years, female, married

That video resonated with me the most. Just like ASHA Didi (sister) explained to me, in that video, she (the lady in the video) explained to another person who used to be troubled by her husband’s problems, such as drinking and not saving money. She explained how to manage your life, like how to save, to do something and not stay idle.

21 years, female, married

Another theme some participants reported was that they could use the chatbot at times convenient to them, for example, during their free time, while others specifically made time for it and integrated it into their daily routines.

When I had free time, I would use it. So, whenever I was bored, I used to open (the video on) social interaction and watch that again. So, I used to watch as much as I liked and then close it later.

42 years, male, married

...I had to cook food, my elder daughter would go to school, and I would go to the shop. I would watch (the videos) there. I would (also) watch while sitting at home in the evening.

30 years, female, married

Many participants mentioned that the chatbot could also benefit other people with stress and related health issues, and some had even shared it with their family and friends.

It (Chatbot) should also be given to those who cannot sleep, it should also be given to those who live in a lot of tension, whose mind will not be able to function, and it must be given to those suffering from depression.

22 years, female, married

I showed it to my friends and others, and they said it (chatbot) is good. They said if they know anyone going through these problems, they will tell them about this (chatbot) so they can fix their problems.

21 years, female, married

#### How PracticePal Complemented the Delivery of the HAP by ASHAs

ASHAs reported that PracticePal improved their ability to communicate and explain therapy messages clearly, reducing their anxiety about delivering incorrect information. They reported that the videos on the chatbot aided them in communicating messages more efficiently.

The program (use of PracticePal) is really good. We have got a lot of help from this for ourselves, for counselling and for the patients as well. The videos worked better for places where we weren’t able to tell the patient. People usually don’t talk so much; sometimes we also used to hesitate because we feared we would say something bad.

FGD with ASHAs

The books we gave them to read sometimes she (patient) used to read them, and sometimes she didn’t. Yes, the mobile was better… She said reading was difficult for her, but she understood better by watching videos. It refreshed her mind.

FGD with ASHAs

ASHAs also appreciated some of the key features of PracticePal. For example, they found that the PracticePal avatar made it easier for patients to relate to the chatbot and improved their engagement in therapy. Some appreciated the feature that provided a summary of patient engagement, as it helped them feel more prepared for upcoming sessions and facilitated discussions with patients during the sessions.

She (chatbot) used to come wearing a saree; they (patients) used to say, “Didi, she looks like you, and she is like you.

FGD with ASHAs

We used to get a summary sheet that would come to us. Through that, we could come to know whether the patient is really doing any activity or not. If we asked the people using the booklet, they would tell us that they did, but how do we know whether they did it or not.

FGD with ASHAs

ASHAs reported encountering resistance from patients’ family members when visiting homes to deliver counseling sessions. These family members often questioned the purpose of the visits and the need for counseling, with some even implying that the ASHAs might think their relatives were “crazy.” In such cases, the chatbot allowed patients to receive therapy content on their mobile phones, as family members had no problems with the patients’ phone use. Thus, the chatbot was instrumental in bypassing the stigma and resistance associated with in-person counseling sessions.

#### How PracticePal Helped the Recovery Process

Many participants reported that the chatbot’s content, particularly the videos, motivated them to engage in behavior activation, interact more, and solve problems, and that these activities led to improvements in their mood.

It (chatbot) said that if you are living under stress, then go out for a walk, look at others, try to understand their point of view very well, just do not stay alone, do not feel stressed. The videos helped me understand. I felt better after seeing it. Earlier I did not do anything, after seeing it I started doing everything. I sit (with others), I work, I feel good in everything.

22 years, female, married

Earlier, I used to not talk about my problems, I just used to say that there is tension, there is tension, but used to not say that I have debt, I don’t have anyone (family). So, I used to not tell it to anyone, it was like a secret… But they (videos) said that if there is any problem then we have to share it. Have to meet people, have to talk, and have to sit. So, I liked this that slowly I told it to everyone then all of them motivated me, that everything will be all right by the time, it just takes some time. So, through this I got that I used to talk, before I used to hesitate.

30 years, female, married

Participants also mentioned that other chatbot features, such as reminders and mood charts, were useful in completing the homework tasks given by ASHAs during the counseling sessions. These tools gave them timely reminders, helped them track their mood, made it easier to stay on top of the exercises, and enabled them to fit the tasks into their daily routines.

It (chatbot) used to remind me every morning and evening of what I had to do that day. It helped a lot; complete plans used to come. The messages (reminders) told me to do things, like watching videos. If we get daily messages, it uplifts the mood. Everyone should do these activities like socializing, making plans. I really liked it.

21 years, female, married

There was a mood scale in it. We had to rate our mood, like how our mood was. So, in the beginning, my mood was like, I would give it a 5 or 6, but gradually, my mood improved. I started giving it a 10.

21 years, female, married

#### Challenges Faced With Using PracticePal

Although there was almost full internet coverage in the study districts, participants sometimes faced issues with slow bandwidth and weak cellular network indoors. While most features of the chatbot worked over low bandwidth, accessing multimedia content faced difficulties. Poor weather conditions, such as rain and cloudy skies, further affected internet connectivity. Some participants had difficulty understanding the flow of PracticePal and choosing from the various menu options provided during the chatbot’s conversation. Anticipating this, the study team had provided them with user manuals, which had screengrabs from the chatbot and clear instructions on the options to choose. Yet, ASHAs had to initially guide the participants in using and navigating the chatbot features during their sessions.

Yes, in the beginning, there was difficulty in using it (chatbot). But when the ASHA would visit for each session, she would start it (chatbot on the mobile) and ask me to read the book (user manual). I read it and understood everything.

22 years, female, married

Initially I had trouble understanding it (chatbot), understanding what to see. Then Didi (ASHA) helped me, but I was not able to understand it right away.

42 years, male, married

Some participants felt that people from different age groups might find it difficult to use the chatbot due to their unfamiliarity with technology. They felt that older individuals or those with less exposure to digital tools might especially struggle to engage with it. It was also pointed out that the chatbot may not be suitable for those who cannot read, limiting its benefits for people with low literacy. It was suggested that the text content be replaced with explanatory videos.

I mean, I am young, so I can do it (use the mobile phone), but there are elders who can't. ASHA Didi should explain to them.

21 years, female, married

Everything is written in it (chatbot). I can read it. But for someone who cannot read, for them it will be easier to follow a video on how to do it (use the chatbot).

22 years, female, married

Some participants felt that the videos provided through the chatbot were repetitive, which led to boredom over time. They appreciated the usefulness of the videos but expressed a strong preference for new content to maintain their interest.

…when something new came up, they would watch it with interest. But it was repeating the same thing, so they were losing interest.

FGD with ASHAs

...they used to say it's the same thing, it's the same thing. So, something new should be added, a new story or improvement from a patient's story. That would benefit them more. [FGD with ASHAs]

## Discussion

This study described the development of PracticePal, a chatbot designed to support the delivery of the HAP, which is a frontline worker–delivered behavioral activation-based psychological treatment for depression, and assessed its feasibility and acceptability in a rural Indian community. The treatment was delivered by ASHAs, community health workers in rural India, to members of their community who had been screened for depression. The chatbot performed multiple roles, particularly reinforcing the knowledge and skills learned in therapy sessions and promoting patient engagement between sessions. Our findings indicate that patient engagement with the chatbot was high and remained consistently strong throughout the duration of the psychological treatment. Participants especially appreciated the reminder feature, which prompted them to complete their planned activities; the multimedia content, which reinforced the key messages from therapy sessions; and the mood monitoring feature. The use of the chatbot improved the overall treatment experience for patients, and the findings are consistent with those of other chatbot studies [36,37].

Our findings of high levels of engagement, acceptability, and feasibility, and the perceived benefits of the chatbot are likely the result of the study design incorporating feedback from both frontline providers and patients with depression. As our results indicate, there was a higher frequency of engagement among female participants than male participants. One of the reasons for this could simply be that there were more female participants than male participants. There was no significant difference observed in the times of the day male and female participants interacted with the chatbot. In addition, it is possible that the higher engagement with the chatbot among female participants than male participants was influenced by differences in smartphone ownership. Women were less likely to own personal smartphones and thus may have used the study-provided phones more extensively, whereas men who owned smartphones may have divided their attention between their personal phones and the study device. We intend to examine this gender-specific engagement pattern further in future studies through a real-world and adequately powered design, wherein participants will access the chatbot using their own smartphones. We note that our results differ from those of some prior studies, which observed that digital tool usage significantly declined over time, typically due to lack of personalization, privacy concerns, trustworthiness, and user interface issues [38,39]. Specifically, we found that our study demonstrated a remarkably high retention rate, with 28 of 30 (93%) participants continuing to engage with the chatbot throughout the average 60-day therapy period. This exceptional engagement can likely be attributed to the chatbot’s role as a complementary therapy aid to in-person NSP-delivered therapy. We suggest 4 key factors that may have contributed to the sustained engagement with PracticePal. First, PracticePal was not designed to replace human interaction but to complement the therapeutic experience without replacing the human connection. The interactive multimedia content further enhanced the experience, reinforcing therapeutic messages in a way that was both engaging and enjoyable for patients. Second, the provider (ie, ASHAs) played a crucial role in reinforcing the use of the chatbot, regularly reminding patients to track their activities and access the content. This was facilitated by a summary sheet that was provided to the ASHAs prior to each session. This sheet detailed the participant’s frequency of engagement, activity completion, and mood reports. The regular reinforcement of participants by the ASHAs helped establish the chatbot as a natural extension of the therapy, making it more acceptable and valuable to patients. It became part of a patient’s routine rather than an external or unfamiliar tool. In comparison to using a paper workbook, which was the original format of the HAP, the chatbot was more readily accessible due to the ubiquitous presence of smartphones, which enabled engagement on demand, and the ease of navigation through its features. Third, the chatbot’s avatar was designed based on local stakeholder input, resembling ASHAs themselves, which helped create a sense of trust and relatability among patients. Finally, the chatbot was embedded in a widely used messaging platform, as opposed to a stand-alone app that has no other purpose.

One of the key strengths of our study was that the chatbot was co-designed with stakeholders and was promoted by frontline health workers who were already embedded in the community, enhancing its credibility and reach. Rather than functioning as a standalone digital tool, the chatbot acted as an aid for frontline health workers, improving the quality of therapy provided. The use of multimedia content increased engagement and buy-in from users, who appreciated the clear and effective messaging. Our study has several limitations. First, the small sample size of the pilot limits the generalizability of the findings. Second, participants in the study were provided with smartphones and data recharges to engage with the chatbot. This raises concerns about accessibility, as individuals without smartphones would be unable to use the chatbot during therapy, potentially limiting its reach to those who already have access to such technology. However, the penetration of smartphones is already very high and rapidly rising across India. Third, the lack of a comparator arm makes it impossible to assess the incremental efficacy of PracticePal added to the HAP compared to the HAP alone. Both the baseline and endline PHQ-9 assessments were conducted by an independent researcher. Baseline assessments were conducted face-to-face, while endline assessments were conducted via phone survey. The use of independent assessors helped minimize the risk of provider bias. However, we acknowledge that the face-to-face format of the baseline assessment may have introduced a degree of social desirability bias, as participants may have felt less comfortable disclosing symptoms in person. In contrast, the phone-based endline assessment may have offered a greater sense of privacy, thereby reducing, though not entirely eliminating, the likelihood of socially desirable responses. Although the HAP is an evidence-based treatment with a robust effect size, future research should involve adequately powered, multi-arm randomized controlled trials to compare the outcomes of the HAP alone, the HAP with the chatbot as a therapy aid, and the chatbot as a standalone intervention.

We also note that the PracticePal chatbot itself may benefit from further enhancements. First, while Telegram was used during the pilot phase due to lower costs, we recognize that WhatsApp is more widely used in India. For future scaling efforts, deploying PracticePal on WhatsApp could enhance accessibility and reach. Second, this chatbot was engineered before the widespread availability of generative artificial intelligence (AI) technologies. The chatbot used in this study was rule-based, and while it was effective, the integration of more advanced AI technologies could offer greater personalization and engagement for users. Future versions of PracticePal could integrate more AI-powered conversational tools to further enhance patient outcomes. Third, PracticePal’s reliance on text-based interactions presented challenges for participants with limited literacy skills. This highlights the need for incorporating audio-to-text capabilities, voice commands, or more video content to enhance accessibility for users with varying literacy levels. Future iterations of the chatbot should incorporate these features to ensure broader usability and inclusivity, particularly in low-literacy settings. Lastly, this study’s findings should be interpreted with caution due to the small sample size, which may limit the generalizability of the results and reduce the statistical power to detect differences in chatbot engagement metrics.

Although there is substantial evidence supporting the effectiveness of mobile platform–delivered digital interventions for improving health outcomes and service utilization in high-income countries, most of these interventions seek to replace a human provider, and much of the evidence is from high-income settings [40]. Our experience reported in a rural Indian community shows that CAs like the PracticePal chatbot, which complement rather than replace human interactions, are highly acceptable to patients with little prior experience of mental health care and can facilitate the delivery of psychosocial interventions by frontline workers. The rapid increase in smartphone penetration across all regions, not least in India, makes the deployment of such chatbots highly feasible.

The PracticePal chatbot is feasible and acceptable as a therapy aid to enhance the quality of NSP-delivered psychosocial interventions in low-resource settings. Its promising potential to improve mental health outcomes underscores the importance of further research to refine and expand the application of chatbot interventions, especially after the advent of generative AI.

Looking ahead, it would be valuable to assess the effectiveness of such chatbots in definitive trials and to assess the efficacy, effectiveness, and cost-effectiveness of chatbots, such as PracticePal, in comparison with traditional treatment options, including as a standalone intervention. Moreover, research should explore the optimal integration of chatbots into broader mental health programs across different settings, addressing potential implementation challenges and scalability.

## Appendix

Multimedia Appendix 1 Multimedia content made available through the chatbot.

Multimedia Appendix 2 PracticePal conversational workflow.

Multimedia Appendix 3 PracticePal avatar and expressions.

Multimedia Appendix 4 Screengrabs of the PracticePal chatbot interface.

## Abbreviations

AI: artificial intelligence
ASHA: accredited social health activist
CA: conversational agent
FGD: focus group discussion
HAP: Healthy Activity Program
IDI: in-depth interview
NSP: nonspecialist provider
PHQ-9: Patient Health Questionnaire-9

## Data Availability

The datasets generated or analyzed during this study are available from the corresponding author upon reasonable request.
