# Supplementary material for: A Conversational Agent (PracticePal) to Support the Delivery of a Brief Behavioral Activation Treatment for Depression in Rural India: Development and Pilot-Testing Study
Source: JMIR Form Res. 2025 Aug 29;9:e73563. doi: 10.2196/73563 (PMC12432468; doi:10.2196/73563)
Supplement: Multimedia Appendix 1 [file formative_v9i1e73563_app1.pdf]

MULTIMEDIA APPENDIX 1: Multimedia content made available through the chatbot

Sr no	Topic	Media type	Description
1	Mental Health	1 Video & 1 flyer	• A video on understanding emotions and mental health.<br>• A flyer on myths and facts about mental health.
2	Depression	3 Videos	• A video explaining depression and its prevalence.<br>• A video on the common misconceptions about depression.<br>• A video on the symptoms of depression.
3	Introduction to Healthy Activity Program (HAP)	2 Videos	• A video on the connection of how our stressors affect our mind, body and behaviour (HAP model).<br>• A video on the importance of HAP activities/homework in treatment.
4	Communicating issues with someone close to you	1 Video	• A video on how to express our thoughts and feelings effectively with someone close to us.
5	Overthinking	1 Video	• A video on how to cope with negative/ anxious thoughts.
6	Structured problem solving	1 Video	• A video on a problem-solving technique that can be implemented across issues clients face.
7	How to sleep well	3 flyers	• 1 flyer on how to have good quality sleep.<br>• 1 flyer on tips to implement when having difficulty sleeping.<br>• 1 flyer on do’s and dont’s before bedtime.

8	Breathing techniques to relax	1 Video	• A video on a breathing technique that can help alleviate stress and anxiety.
9	Effect and reduction of alcohol consumption	1 Video & 1 flyer	• A video on the harmful effects of alcohol consumption on one's physical and mental health and well-being.<br>• A flyer on tips to reduce alcohol consumption.
10	Effect and reduction of tobacco consumption	2 Videos	• A video on the effect of tobacco on physical and mental health and the benefits of reducing consumption to the body.<br>• A video on methods that can be used to reduce smoking.
11	Helping those facing domestic violence	1 Video	• A video on domestic violence that highlights its effect on one's mental health and emotional wellbeing. The video provides tips and resources one can avail of or provide to those who face domestic violence.
12	Dealing with anxiety	1 Video	• A video on anxiety and its symptoms and how it can affect one's health.
13	Frequently asked questions about medication for stress	1 Video	• A video on:<br>○ How medication can help<br>○ How long it takes for medication to take effect<br>○ How long medication must be taken<br>○ Side effects of taking medication
