# Supplementary figures and images for: A Conversational Agent (PracticePal) to Support the Delivery of a Brief Behavioral Activation Treatment for Depression in Rural India: Development and Pilot-Testing Study
Source: JMIR Form Res. 2025 Aug 29;9:e73563. doi: 10.2196/73563 (PMC12432468; doi:10.2196/73563)

## MULTIMEDIA APPENDIX 2: PracticePal Conversational Workflow

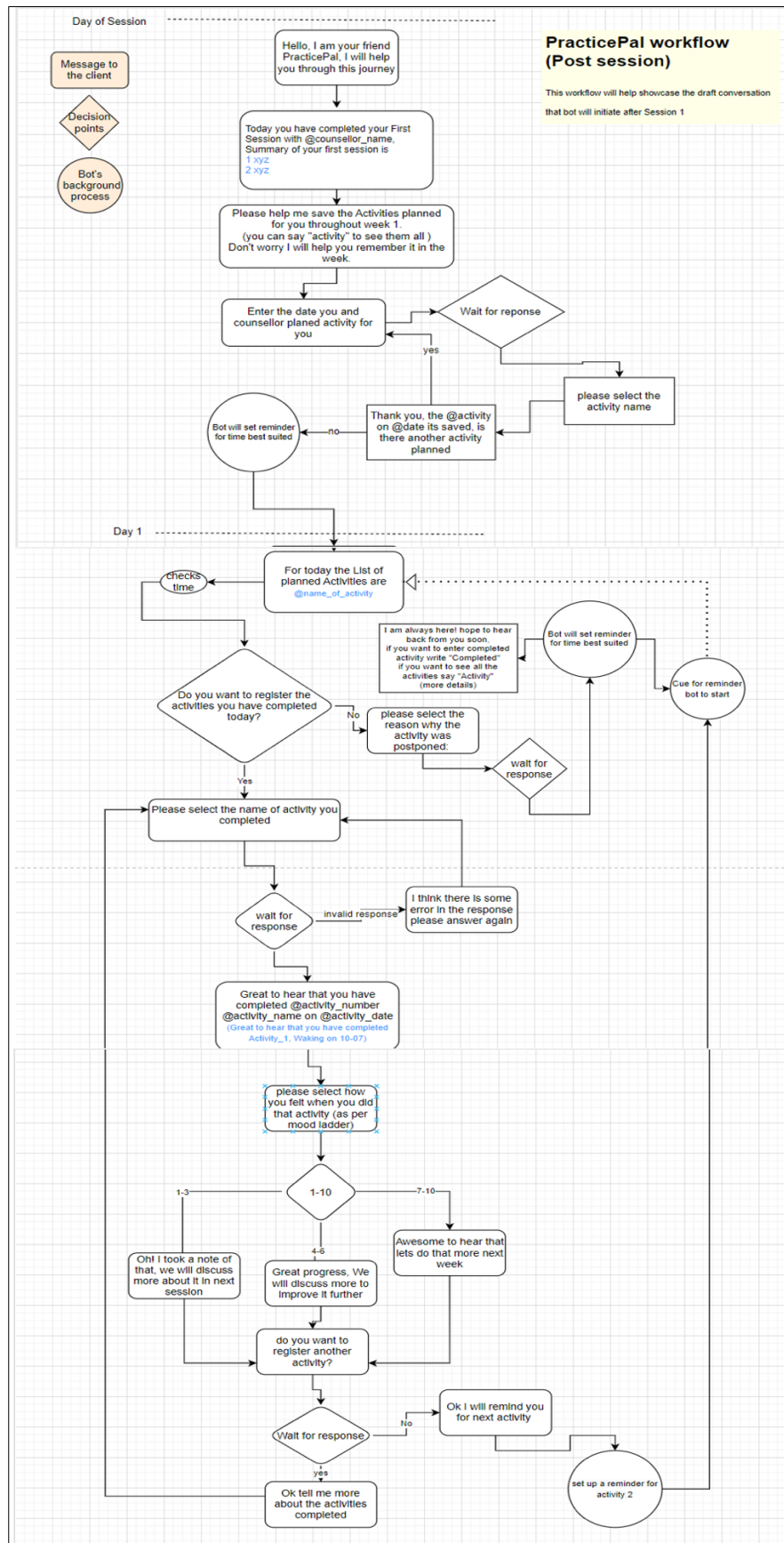

Supplement: Multimedia Appendix 2 [file formative_v9i1e73563_app2.pdf]

### Multimedia Appendix 3: PracticePal avatar and expressions

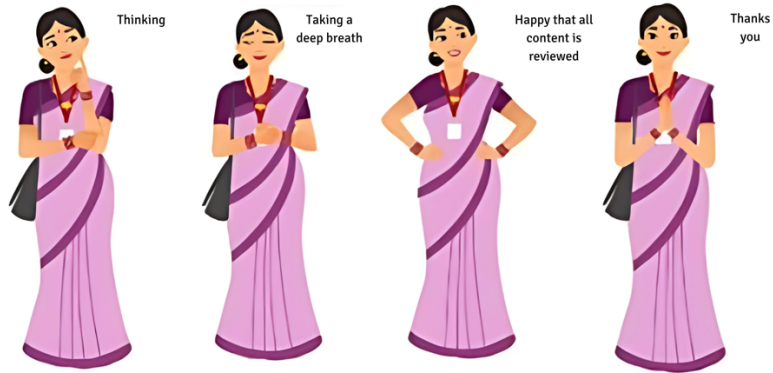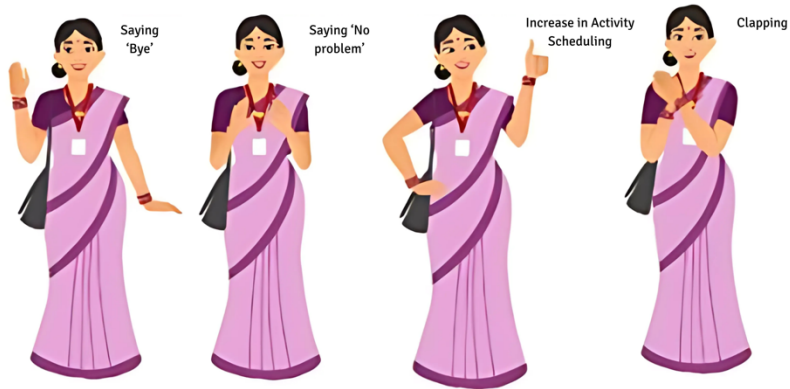

Supplement: Multimedia Appendix 3 [file formative_v9i1e73563_app3.pdf]
