# Supplementary material for: A Conversational Agent (PracticePal) to Support the Delivery of a Brief Behavioral Activation Treatment for Depression in Rural India: Development and Pilot-Testing Study
Source: JMIR Form Res. 2025 Aug 29;9:e73563. doi: 10.2196/73563 (PMC12432468; doi:10.2196/73563)

Multimedia Appendix 4 : Screengrabs of PracticePal interface

Welcome greeting by the chatbot on typing hi or hello.

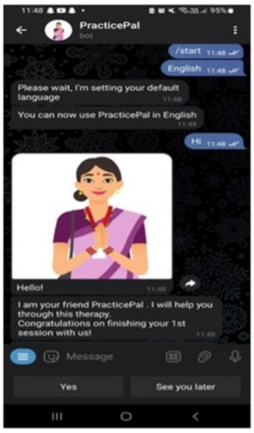

Chatbot offering a choice of learning resources to the user

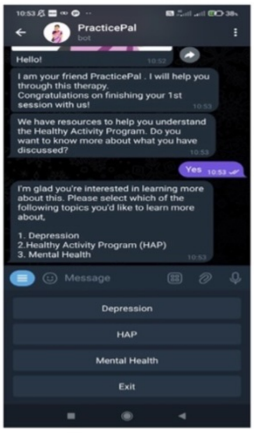

Chatbot offering the choice of 'activity scheduling' as part of homework tasks

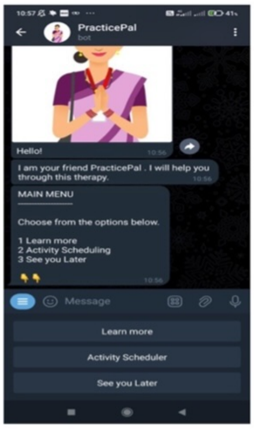

Chatbot inviting user to record activity status

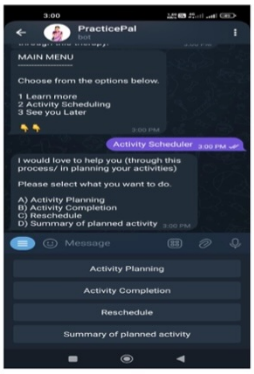

Chatbot offering a choice of behaviour activation tasks

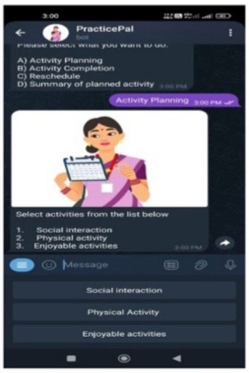

Chatbot inviting the user to record their mood.

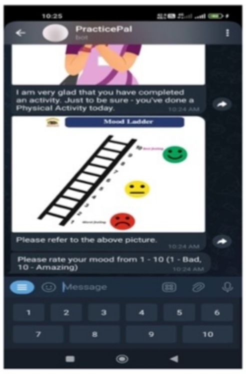

Supplement: Multimedia Appendix 4 [file formative_v9i1e73563_app4.pdf]
